# Impaired fasting glucose is associated with increased perioperative cardiovascular event rates in patients undergoing major non-cardiothoracic surgery

**DOI:** 10.1186/1475-2840-10-63

**Published:** 2011-07-14

**Authors:** Murat Biteker, Akin Dayan, Mehmet M Can, Erkan İlhan, Funda S Biteker, Ahmet Tekkeşin, Dursun Duman

**Affiliations:** 1 Haydarpaşa Numune Education and Research Hospital, Department of Cardiology, Istanbul, Turkey; 2 Haydarpaşa Numune Education and Research Hospital, Department of Family Medicine, Istanbul, Turkey; 3 Gata Haydarpaşa Military Education and Research Hospital, Department of Cardiology, Istanbul, Turkey; 4 Siyami Ersek Cardiovascular Surgery Center, Department of Cardiology, Istanbul, Turkey; 5 Kartal Dr Lutfi Kırdar Education and Research Hospital, Department of Infectious Diseases and Clinical Microbiology, Istanbul, Turkey

**Keywords:** noncardiothoracic surgery, preoperative glucose levels, cardiovascular complications

## Abstract

**Background:**

Diabetes mellitus (DM) is a well-established risk factor for perioperative cardiovascular morbidity and mortality in patients undergoing noncardiac surgery. However, the impact of preoperative glucose levels on perioperative cardiovascular outcomes in patients undergoing nonemergent, major noncardiothoracic surgery is unclear.

**Methods and Results:**

A total of 680 patients undergoing noncardiothoracic surgery were prospectively evaluated. Patients older than 18 years who underwent an elective, nonday case, open surgical procedure were enrolled. Electrocardiography and cardiac biomarkers were obtained 1 day before surgery, and on days 1, 3 and 7 after surgery. Preoperative risk factors and laboratory test results were measured and evaluated for their association with the occurrence of in-hospital perioperative cardiovascular events. Impaired fasting glucose (IFG) defined as fasting plasma glucose values of 100 to 125 mg/dl; DM was defined as fasting plasma glucose ≥ 126 mg/dl and/or plasma glucose ≥ 200 mg/dl or the current use of blood glucose-lowering medication, and glucose values below 100 mg/dl were considered normal. Plasma glucose levels were significantly higher in patients with perioperative cardiovascular events (n = 80, 11.8%) in comparison to those without cardiovascular events (131 ± 42.5 *vs *106.5 ± 37.5, p < 0.0001). Multivariate analysis revealed that patients with IFG and DM were at 2.1- and 6.4-fold increased risk of perioperative cardiovascular events, respectively. Every 10 mg/dl increase in preoperative plasma glucose levels was related to a 11% increase for adverse perioperative cardiovascular events.

**Conclusions:**

Not only DM but also IFG is associated with increased perioperative cardiovascular event rates in patients undergoing noncardiothoracic surgery.

## Background

Patients undergoing major noncardiac surgery are at high risk of cardiovascular complications [1]. Diabetes mellitus (DM) and hyperglycemia are risk factors for adverse perioperative cardiac and noncardiac events [2-5]. In patients with known DM, the risk of atherosclerotic cardiovascular disease is increased with increasing plasma glucose concentration. Each 1% increase in glycated haemoglobin (HbA 1c) level was associated with a 14% increase in the incidence of fatal and nonfatal myocardial infarction [6]. However, more recently the emphasis has shifted from diabetes to new-onset hyperglycemia [7]. Pre-diabetes represents a metabolic stage intermediate between normal glucose hemostasis and DM [7]. Although DM has been recognized as an independent predictor of perioperative cardiovascular outcomes, the prognosis of nondiabetic patients with impaired glucose regulation is not clear. The relationship between preoperative glucose levels and perioperative adverse cardiovascular events in noncardiac surgical patients is not evaluated in prospective, randomized studies. This study was conducted to elucidate the association between preoperative glucose levels with perioperative cardiovascular events in patients undergoing major noncardiac, nonvascular surgery.

## Methods

### Study design and patient population

This study was conducted during a 1-year period in 2010 at Haydarpaşa Numune Education and Research Hospital. This hospital is a tertiary medical center in Istanbul/Turkey. After approval by the institutional review board, 680 consecutive patients, aged 18 yr or older, presenting for anesthetic and noncardiothoracic, nonvascular surgery, were included in the study. Written informed consent was obtained from each patient before entry into the study. Patients presenting for surgery requiring only local anesthesia or monitored anesthesia care and day case surgical procedures were excluded. Emergent surgical cases and the patients with an American Society of Anesthesiologists (ASA) classification 5 (moribund, not expected to live 24 h irrespective of operation) were also excluded [8]. The type of surgery was classified and categorized according to the surgical risk, determined using the American College of Cardiology/American Heart Association (ACC/AHA) classification [9]. Intermediate-risk surgery was defined as surgery with a cardiac risk of 1-5%, and included head and neck surgery, intraperitoneal and intrathoracic surgery, orthopaedic and prostate surgery. In our study patients, major gastrointestinal surgery (laparotomy, advanced bowel surgery, gastric surgery), major gynaecological cancer surgery (abdominal hysterectomy and oopharectomy for cancer), and major open or transurethral urological surgery (cystectomy, radical nephrectomy, total prostatectomy), head and neck surgery, hip or knee arthroplasty were included. Intrathoracic surgery is not performed in our institution.

Risk assessment, preoperative preparation and drug therapy and postoperative follow-up were completed according to current ACC/AHA guidelines [9]. For each patient, preoperative risk factors of morbidity and mortality, patients' characteristics, preoperative medication and intraoperative data were prospectively evaluated. In the preoperative period, classification of the ASA was used as a composite index of a patient's general status [8]. The Revised Cardiac Risk Index (RCRI) was used for prediction of cardiac risk based on six prognostic factors: high-risk type of surgery (defined as intraperitoneal, intrathoracic, or suprainguinal vascular procedures), ischemic heart disease, congestive heart failure, history of cerebrovascular disease, insulin therapy for diabetes, and preoperative serum creatinine > 2.0 mg/dl [10]. Each of the prognostic factors assigned as one point. Anesthetic management, monitoring, and other aspects of perioperative management were at the discretion of the attending physician.

Electrocardiography (12-lead) and cardiac biomarkers (creatine kinase-MB and troponin I) were evaluated 1 day before surgery, immediately after surgery, and on post-operative days 1, 3 and 7.

All patients were fasting ≥ 8 h and were scheduled to have their surgery in the morning. Baseline glucose measurements were obtained during preoperative assessment at a central laboratory. Blood samples were obtained using venipuncture with minimal stasis. Glucose was enzymatically determined using the Hexokinase method (Boehringer, Mannheim, Germany). Information was obtained on history of diabetes and use of blood glucose-lowering treatment. Impaired fasting glucose (IFG), and DM were defined according to the American Diabetes Association (ADA) criteria [7];

• Patients with a known history of DM, a preoperative fasting blood glucose level ≥ 126 mg/dl, or 2 or more outpatient random blood glucose levels ≥ 200 mg/dl or the current use of blood glucose-lowering medication were classified into the DM group,

• Impaired fasting glucose defined as fasting plasma glucose values of 100 to 125 mg per/dl and,

• Fasting glucose values below 100 mg per/dl were considered normal.

Our protocol for peri-operative blood glucose control was based on the Alberti Regimen which involves the use of an intravenous infusion of a pre-mixed bag of glucose-insuline-potassium [11].

### Study end-points

Patients were followed up by the consulting physician until discharge after surgery. The perioperative cardiovascular events were defined as the occurrence of severe arrhythmias requiring treatment, cardiac death (death caused by acute myocardial infarction, significant cardiac arrhythmias, refractory congestive heart failure or as a death occurring suddenly without another explanation), acute heart failure, acute coronary syndrome (nonfatal acute myocardial infarction or unstable angina), pulmonary thromboembolism, nonfatal cardiac arrest, and cardioembolic stroke. Myocardial infarction is diagnosed when the following criteria are present: detection of a rise of troponin with at least one value above the 99th percentile of the upper reference limit together with evidence of myocardial ischemia as recognized by at least one of the following: symptoms of ischemia, ECG changes of new ischemia or development of pathologic Q waves, or new regional wall motion abnormality according to the universal definition of myocardial infarction [12]. This single-center cohort study was approved by the ethical committee at the authors' institution.

### Statistical Analysis

Data were analyzed using SPSS for Windows (version 15, SPSS Inc, Chicago, Illinois). The continuous variables were expressed as mean ± standard deviation and were compared between groups by two-tailed Student t test. Nonparametric tests were also used when necessary (Mann-Whitney U test). Fisher Exact (Chi-square) test was used in comparison of categorical variables. For all analyses, p < 0.05 was considered statistically significant. Univariate and multivariate logistic regression analyses were applied to determine crude and adjusted odds ratios (ORs) and 95% confidence intervals (CIs) for the relation between preoperative glucose levels and perioperative cardiovascular adverse events.

## Results

Seven hundred patients were initially included in the study. Eleven patients were excluded because of insufficient data and nine patients were excluded because of an emergency operation. The baseline clinical and demographic characteristics of the patients with normal glucose levels (*n *= 344, 50.6%), IFG (*n *= 132, 19.4%) and DM (*n *= 204, 30%) are presented in Table 1, and perioperative characteristics are presented in Table 2. There were no significant difference regarding the type of surgical procedure, age, gender, and body mass index between patients with normal glucose levels, impaired glucose regulation, and diabetes.

**Table 1:** Baseline clinical and demographic characteristics

		All patients (n = 680)	Normal glucose (n = 344)	Impaired fasting glucose (n = 132)	Diabetes mellitus (n = 204)	*P *value
**Age**		65,4 ± 14	62,5 ± 15,6	68,9 ± 11,9	67,8 ± 11,3	0,328
**Male**		358(52,6)	173(50,3)	66(50)	119(58,3)	0,151
**Body mass index**		28,3 ± 12,2	27,6 ± 5,5	27,4 ± 3,8	30,2 ± 20,8	0,146
**ASA status**	1	117(17,2)	88(25,6)	18(13,8)	**0**	< 0,001
	2	344(50,6)	182(52,9)	76(57,6)	**97(47,5)**	
	3	166(24,4)	54(15,7)	27(20,5)	85(41,7)	
	4	53(7,8)	20(5,8)	11(8,3)	22(10,8)	
**Hypertension**		375(55,1)	152(44,2)	79(59,8)	144(70,6)	< 0,001
**Current smoking**		100(14,7)	51(14,8)	17(12,9)	32(15,7)	0,66
**Coronary artery disease**		182(26,8)	64(18,6)	29(22)	89(43,6)	< 0,001
**Heart failure**		69(10,1)	20(5,8)	16(12,1)	33(16,2)	< 0,001
**History of CVA**		65(9,6)	24(7)	16(12,1)	25(12,3)	0,068
**Atrial fibrillation**		82(12,1)	43(12,5)	13(9,8)	26(12,7)	0,683
**NYHA functional class**	1	426(62,6)	233(67,7)	85(64,4)	108(52,9)	< 0,001
	2	235(34,6)	108(31,4)	45(34,1)	82(40,2)	
	3	18(2,6)	3(0,9)	2(1,5)	13(6,4)	
	4	1(0,1)	0(0)	0(0)	1(0,5)	
**Revised cardiac risk index**	0	95(14)	63(18,3)	19(14,4)	13(6,4)	< 0,001
	1	313(46)	187(54,4)	57(43,2)	69(33,8)	
	2	195(28,7)	75(21,8)	45(34,1)	75(36,8)	
	3	70(10,3)	19(5,5)	10(7,6)	41(20,1)	
	4	7(1)	0(0)	1(0,8)	6(2,9)	

Abbreviations: ASA = American Society of Anesthesiologists; CVA = cerebrovascular accident: NYHA = New York Heart Association. Values are given as mean ± SD or number (percentage).

**Table 2:** Perioperative characteristics

		All patients (n = 680)	Normal glucose (n = 344)	Impaired fasting glucose (n = 132)	Diabetes mellitus (n = 204)	*P *value
**Beta-blocker**		157(23,1)	61(17,7)	33(25)	63(30,9)	0,002
**Calcium inhibitor**		99(14,6)	51(14,8)	16(12,1)	32(15,7)	0,651
**ACE inhibitor**		180(26,5)	75(21,8)	37(28)	68(33,3)	0,011
**Furosemid**		21(3,1)	10(2,9)	7(5,3)	4(2)	0,216
**Thiazid**		22(3,2)	10(2,9)	4(3)	8(3,9)	0,801
**Sprinolactone**		20(2,9)	8(2,3)	6(4,5)	6(2,9)	0,439
**Aspirin**		146(21,5)	47(13,7)	27(20,5)	72(35,3)	< 0,001
**Statin**		67(9,9)	17(4,9)	7(5,3)	43(21,1)	< 0,001
**Digoxin**		25(3,7)	10(2,9)	7(5,3)	8(3,9)	0,450
**Anticoagulant**		31(4,6)	11(3,2)	9(6,8)	11(5,4)	0,188
**ARB**		60(8,8)	25(7,3)	9(6,8)	26(12,7)	0,061
	**General**	288(42,4)	143(41,6)	58(43,9)	87(42,6)	
	**Urology**	131(19,3)	70(20,3)	25(18,9)	36(17,6)	
	**Plastics**	32(4,7)	17(4,9)	7(5,3)	8(3,9)	
**Type of surgery**	**Gynecological**	33(4,9)	18(5,2)	5(3,8)	10(4,9)	0,724
	**Orthopedic**	160(23,5)	77(22,4)	35(26,5)	48(23,5)	
	**Ophthalmologic**	9(1,3)	4(1,2)	2(1,5)	3(1,5)	
	**Neurological**	19(2,8)	11(3,2)	1(0,8)	7(3,4)	
	**Ear/nose/throat**	8(1,2)	4(1,2)	2(1,5)	2(1)	
**Triglyceride (mg/dl)**		143,4 ± 75,9	141,3 ± 85,5	137,9 ± 55,8	150,5 ± 69,4	0,182
**High density lipoprotein (mg/dl)**		45,6 ± 17,4	46,4 ± 18,4	45 ± 15,7	44,6 ± 16,7	0,323
**Low density lipoprotein (mg/dl)**		108,8 ± 32,7	107,4 ± 32,1	114,3 ± 36,3	107,6 ± 31	0,148
**Creatinine (mg/dl)**		1,2 ± 1,4	1,2 ± 1,6	1,2 ± 1,2	1,2 ± 1,1	0,163
**Fasting glucose (mg/dl)**		109,4 ± 38,7	86,8 ± 8,6	109,6 ± 7,3	147,6 ± 50,1	< 0,001

Abbreviations: ACE = Angiotensin-converting enzyme inhibitor; ARB = angiotensin II-receptor blocker. Values are given as mean ± SD or number (percentage).

Prevalence of coronary/peripheral artery disease, hypertension, and congestive heart failure was higher in diabetic patients compared with patients who had impaired glucose regulation. Patients with impaired glucose regulation had also higher prevalence of coronary/peripheral artery disease, hypertension, and congestive heart failure compared with normoglycemic patients. New York Heart Association functional capacity, ASA status, and RCRI were worse in patients with DM compared to patients with impaired glucose regulation, and in patients with impaired glucose regulation compared to normoglycemic patients. Preoperative use of beta blocker, angiotensin-converting enzyme inhibitors, statins and aspirin was higher in patients with DM compared to patients with impaired glucose regulation, and in patients with impaired glucose regulation compared to patients with normal glucose levels.

### Preoperative glucose levels and perioperative cardiovascular events

**Figure 1:**
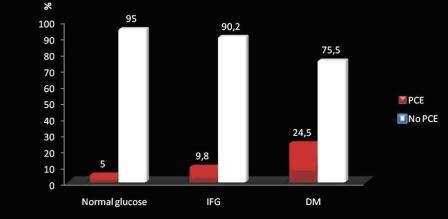
**Perioperative adverse cardiovascular events (PCE) in patient groups**.

A total of 80 patients (11.8%) had perioperative cardiovascular complications (Table 3). Fifty of the patients with DM (24.5%), 13 of the patients with impaired glucose tolerance (9.8%), and 17 of the patients with normal glucose levels (5%) had adverse perioperative cardiovascular events (*p *< 0.001) (Figure 1). Distribution of adverse events were similar in patients with normal glucose levels, impaired fasting glucose levels, and DM (p = 0.51) (Table 3). Median preoperative plasma glucose levels were significantly higher in patients with adverse perioperative cardiovascular events compared to patients with no cardiovascular events (124 [range 131 ± 42.5] *vs *196 [106.5 ± 37.5], p < 0.001).

**Table 3:** Adverse perioperative cardiovascular events

		All patients (n = 680)	Normal glucose (n = 344)	Impaired fasting glucose (n = 132)	Diabetes mellitus (n = 204)	*P *value
**PCE**	**No**	600(88,2)	327(95)	119(90,2)	154(75,5)	< 0,001
	**Yes**	80(11,8)	17(5)	13(9,8)	50(24,5)	

	**Acute coronary syndrome**	24(30)	6(35,3)	6(46,2)	12(24)	
	**Severe arrhythmia**	12(15)	2(11,8)	1(7,7)	9(18)	
**Distrubition of PCE**	**Nonfatal cardiac arrest**	5(6,3)	1(5,9)	1(7,7)	3(6)	0,512
	**Cardiovascular death**	5(6,3)	1(5,9)	0(0)	4(8)	
	**Cardioembolic stroke**	10(12,5)	1(5,9)	2(15,4)	7(14)	
	**Pulmonary embolism**	7(8,8)	4(23,5)	0(0)	3(6)	
	**Acute heart failure**	17(21,3)	2(11,8)	3(23,1)	12(24)	

Abbreviations: PCE = Perioperative cardiovascular adverse event. Values are given as mean ± SD or number (percentage)

The median plasma glucose levels of patients with adverse perioperative cardiovascular events were significantly different in the three patient groups (148 mg/dl [84-286] in diabetic patients, 114 mg/dl [100-125] in patients with IFG, and 86 mg/dl [73-97] in normoglycemic patients, p < 0.001). Univariate analysis showed a significant association between age, DM, IFG, coronary artery disease, preoperative atrial fibrillation, American Society of Anesthesiologists status, Revised Cardiac Risk Index and perioperative cardiovascular events. On multivariate logistic regression analysis DM and IFG remained as significant variables associated with perioperative cardiovascular complications. Patients with impaired glucose regulation and DM were at 2.1-fold (adjusted OR 2.1 and 95% CI 0.99-4.49; p < 0.04), and 6.4-fold (adjusted OR 6.4 and 95% CI 3.57-11.48; p < 0.001) increased risk for perioperative cardiovascular events, respectively. Every 10 mg/dl increase in preoperative plasma glucose levels was related to a 11% increase for adverse perioperative cardiovascular events.

## Discussion

Our study demonstrated that increased preoperative glucose levels are associated with adverse perioperative cardiovascular events in patients undergoing major noncardiac surgery. Not only DM, but also impaired glucose regulation was associated with an increased incidence of perioperative cardiovascular complications.

Recent data indicate that postprandial hyperglycemia or even impaired glucose tolerance may predispose to progression of atherosclerosis and cardiovascular events [13]. However, the prognostic role of impaired glucose tolerance in noncardiac surgery patients is not fully elucidated. Although DM has been recognized as an independent predictor of perioperative cardiovascular complications in patients undergoing noncardiac surgery only a few studies take preoperative glucose levels into account. The association between preoperative glucose levels and perioperative cardiac and noncardiac complications is, however, controversial. Velanovich [14] found that an abnormal preoperative glucose value was an independent predictor of postoperative bleeding, whereas McKee and Scott demonstrated the association of postoperative complications with any preoperative laboratory abnormalities [15]. Dzankic et al. evaluated the prevalence and predictive value of abnormal preoperative laboratory tests in 544 consecutive patients ≥ 70 yr old who were undergoing noncardiac surgery [16] Abnormal hemoglobin, creatinine, and glucose values were not predictive of postoperative adverse outcomes. Only ASA classification and surgical risk were significant independent predictors of postoperative adverse outcomes.

Feringa et al. investigated whether impaired glucose regulation and elevated glycated HbA 1c levels are associated with increased cardiac ischaemic events in vascular surgery patients [17]. They have measured baseline glucose and HbA 1c levels in 401 vascular surgery patients. Using subjects with normal glucose levels as the reference category, multivariate analysis revealed that patients with impaired glucose regulation and diabetes were at 2.2- and 2.6-fold increased risk of ischaemia, 3.8- and 3.9-fold for troponin release, 4.3- and 4.8-fold for 30-day cardiac events, and 1.9- and 3.1-fold for long-term cardiac events. Patients with HbA 1c > 7.0% were at 2.8-fold, 2.1-fold, 5.3-fold and 5.6-fold increased risk for ischaemia, troponin release, 30-day and long-term cardiac events, respectively. This study demonstrated the value of preoperative glucose and HbA1c in defining perioperative and long-term risk in vascular surgery patients.

Impaired glucose regulation is a term that refers to blood glucose levels that are above the normal range but are not high enough for the diagnosis of DM [7]. This term is used to describe the presence of IFG and/or impaired glucose tolerance, which are intermediate states of abnormal glucose regulation that exist between normal blood glucose levels and DM. Both IFG and impaired glucose tolerance are risk factors for the subsequent development of both DM and cardiovascular disease [18-21]. However, it is unclear whether IFG or impaired glucose tolerance are independent risk factors in the development of cardiovascular complications in patients undergoing noncardiac surgery.

Dunkelgrun et al. showed that the prevalence of undiagnosed impaired glucose tolerance and DM is high in vascular surgery patients. Of 404 patients without histories of impaired glucose tolerance and DM, 26% had impaired glucose tolerance and 11% diabetes [22]. Furthermore, the presence of DM significantly predicted the risk for perioperative cardiac ischemia (OR = 3.2, 95%CI 1.3-8.1). Kuijk et al. also showed that the patients with impaired glucose tolerance had adverse prognosis even worse than that of patients with DM in vascular surgery patients [23]. Noordzij et al. undertook a retrospective case-control study among 904 cases and 1247 controls who underwent noncardiac, nonvascular surgical procedures in the Erasmus MC [24]. In this study, they showed that prediabetes glucose levels had a 3-fold increased cardiovascular mortality and diabetes glucose levels had a 4-fold increased cardiovascular mortality risk. In subjects with prediabetes glucose levels, a continuously increasing risk of perioperative mortality was observed along with increasing glucose concentration and each 1 mmol/l increase was associated with a 19% risk increase for mortality. The results of this study is important to show that prediabetes glucose levels in patients without a history of diabetes were also associated with increased perioperative cardiovascular death. Although our study differs from the Noordzij et al. study with its prospective design, we have also showed that the patients with impaired glucose regulation undergoing noncardiac, nonvascular surgery are at risk for developing cardiovascular complications.

Recently, Hatzakorzian et al. observed fasting hyperglycemia in > 25% of presumably non-diabetic patients presenting for elective surgery [25]. However, they have not evaluated the significance of preoperative IFG on peri-operative cardiovascular outcomes. In the current study, 19.4% of our patients presenting for elective noncardiac surgery had fasting blood glucose levels compatible with IFG, and 30% of the patients had DM. Although the prevalence of DM and IFG is higher than the world wide [26], our population consisted of patients with a mean age of 65 years and undergoing surgery in a tertiary center.

### Study Limitations

Our study did not address the question of whether treatment of hyperglycemia may reduce the high morbidity and mortality associated with hyperglycemia in patients with IFG or DM. The second limitation of our study is that we were not able to determine the percentage of patients with latent DM because of the lack of hemoglobin A1C testing and the lack of follow-up after discharge. Stress- induced hyperglycemia, could have partly contributed to elevated fasting blood glucose levels. However, it was not possible to differentiate between stress-induced perioperative hyperglycemia and blood glucose rise associated with preexisting glycemic disease.

## Conclusions

In conclusion, this study shows that impaired glucose regulation and DM are risk factors for perioperative cardiovascular events in noncardiac surgery patients. The results of our study suggest that screening for glucose dysregulation in these patients should be part of standard preoperative testing. However, the value of screening for dysglycaemia and the benefits of aggressive glucose management require further prospective, randomized studies.

## Competing interests

The authors declare that they have no competing interests.

## Authors' contributions

MB: Performing measurements, writing manuscript.

AD, AT, DD: Performing measurements,discussion of manuscript.

MMC: Discussion of data analysis and manuscript.

EI, FSB: Helping to set up methodology, discussion of data and manuscript.
